# Targeting a Newly Established Spontaneous Feline Fibrosarcoma Cell Line by Gene Transfer

**DOI:** 10.1371/journal.pone.0037743

**Published:** 2012-05-30

**Authors:** Rounak Nande, Altomare Di Benedetto, Pierpaolo Aimola, Flavia De Carlo, Miranda Carper, Charlene D. Claudio, Jim Denvir, Jagan Valluri, Gary C. Duncan, Pier Paolo Claudio

**Affiliations:** 1 Department of Biochemistry and Microbiology, Joan C. Edwards School of Medicine, Marshall University, Huntington, West Virginia, United States of America; 2 McKown Translational Research Institute, Edwards Comprehensive Cancer Center, Huntington, West Virginia, United States of America; 3 Department of Basic and Applied Biology, Faculty of Sciences, University of L’Aquila, L’Aquila, Italy; 4 Department of Biological Sciences, Marshall University, Huntington, West Virginia, United States of America; 5 Martin Veterinary Clinic, Ashland, Kentucky, United States of America; 6 Department of Surgery, Joan C. Edwards School of Medicine, Marshall University, Huntington, West Virginia, United States of America; University of Chicago, United States of America

## Abstract

Fibrosarcoma is a deadly disease in cats and is significantly more often located at classical vaccine injections sites. More rare forms of spontaneous non-vaccination site (NSV) fibrosarcomas have been described and have been found associated to genetic alterations. Purpose of this study was to compare the efficacy of adenoviral gene transfer in NVS fibrosarcoma. We isolated and characterized a NVS fibrosarcoma cell line (Cocca-6A) from a spontaneous fibrosarcoma that occurred in a domestic calico cat. The feline cells were karyotyped and their chromosome number was counted using a Giemsa staining. Adenoviral gene transfer was verified by western blot analysis. Flow cytometry assay and Annexin-V were used to study cell-cycle changes and cell death of transduced cells. Cocca-6A fibrosarcoma cells were morphologically and cytogenetically characterized. Giemsa block staining of metaphase spreads of the Cocca-6A cells showed deletion of one of the E1 chromosomes, where feline p53 maps. Semi-quantitative PCR demonstrated reduction of p53 genomic DNA in the Cocca-6A cells. Adenoviral gene transfer determined a remarkable effect on the viability and growth of the Cocca-6A cells following single transduction with adenoviruses carrying *Mda-7/IL-24* or IFN-γ or various combination of *RB/p105*, *Ras*-DN, IFN-γ, and Mda-7 gene transfer. Therapy for feline fibrosarcomas is often insufficient for long lasting tumor eradication. More gene transfer studies should be conducted in order to understand if these viral vectors could be applicable regardless the origin (spontaneous vs. vaccine induced) of feline fibrosarcomas.

## Introduction

Fibrosarcoma represents 6–12% of all feline tumors [1]. It is a malignant tumor of mesenchymal origin, derived from fibrous connective tissue with the presence of undifferentiated proliferating fibroblasts in a collagen matrix, which normally develops in soft tissues. Fibrosarcoma tumors are significantly more often located at classical vaccine injections sites on cats. An epidemiologic analysis showed correlation between fibrosarcoma and injection sites for leukosis vaccines [2]. The vaccines generally associated with this disease to date have been the adjuvanted rabies and feline leukemia virus vaccines; however, association with non-adjuvanted vaccines has been occasionally reported [2]. More rare forms of spontaneous fibrosarcoma have been described and have been found associated to genetic alterations, such as allelic loss, point mutations and translocations [3], [4].

Surgery is the typical therapeutic treatment, followed if needed by radiotherapy. Despite the effectiveness of this treatment, there is a high neoplasm recurrence on the primary tumor site [5]. Several studies reported the low efficiency of chemotherapy by itself [6], [7]. Due to poor cure rates with surgery alone, the additional use of adjuvant radiation therapy and/or chemotherapy has been under investigation at multiple veterinary cancer centers for the last few years [8], [9]. Recently, it has been suggested that the use of a tri-modal therapeutic approach (radical surgery, radiation therapy and chemotherapy) will very likely be the preferred therapy for this extremely malignant tumor [9]. Other options that are being explored encompass also gene therapy and immunostimolatory gene therapy, which constitute the most promising treatments [5], [10]. The efficiency of proteins able to stimulate immune responses in feline fibrosarcoma has been reported in few recent scientific reports [5], [10]. Recombinant plasmids coding IL-2, IFN-γ, and GM-CSF have been used in phase-I studies using intratumoral magnetofection [11]. Additionally, cats and dogs treated with repeated local injections of engineered histo-incompatible cells secreting high levels of human interleukin-2 (hIL-2) to the tumor site relapse less frequently and survive longer than control animals treated by surgery and radiotherapy alone [12]. Recently, viral vectors expressing feline or human IL2, respectively, were administered to domestic cats and it was shown a 33% reduction of tumor recurrences in cats receiving either human IL2 or feline IL2 [5].

We have isolated and characterized cells cultured from a biopsy of a spontaneous fibrosarcoma taken from a domestic female calico cat. Because the prognosis of such tumors continues to be poor, we have compared and contrasted the effects of single and combinatorial gene transfer using replication deficient adenoviruses expressing *RB/p105*, p130, p53, p18, p19, p21^waf-1^, p27^kip-1^, pTEN, *Mda-7/IL-24*, and *Ras*-DN. We also studied the effects of conditionally replication-competent adenovirus (CRCA) overexpressing immunostimolatory proteins such as *IFN-γ* or *Mda-7/IL-24* and the combination of the above viral vectors to identify potential cellular targets for the treatment of this tumor.

## Materials and Methods

### Clinical Data

A 10-years old domestic calico female cat presented with a rapidly growing lesion on the first digit of the right front paw, which became ulcerated and started bleeding within 3 weeks from the first observation. The tumor formation was surgically removed in March 2007 at the Martin’s Veterinarian Clinic in Ashland, Kentucky and it was histologically diagnosed as a fibrosarcoma that appeared to be completely excised. No chemo- or radiation-therapy was given. Tumor recurred within 15-days from the first operation and appeared to grow at a faster rate, doubling its size in the matter of one week. The cat was amputated of the diseased limb and a biopsy of the recurred tumor was collected in a sterile 50-mL tube containing sterile cold isotonic saline solution. Recurred tumor was sent to a veterinarian pathology laboratory to reconfirm the histological diagnosis.

**Figure 1:**
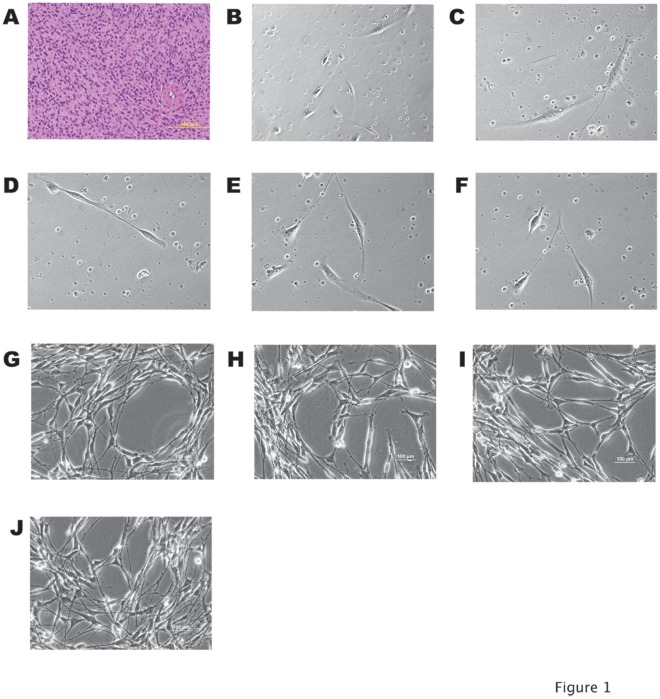
Establishment of a feline fibrosarcoma cell line. (A) Hematoxylin & Eosin staining of the pathologic specimen. Optical microscope 100× magnification of the neoplastic tissue shows cells that appear to have indistinct cell borders, round to oval nuclei with finely stippled chromatin, and small single or multiple nucleoli. (B–F) Phase contrast imaging of the Cocca-1A, -2A, -3A, -6A, and -3B clones after 48 hours of culture (200× magnification). (G) Phase contrast, 200× magnification of the clone 6A after 3 months of continuous culture. (H–J) Phase contrast, 200× magnification of the clone 6A after 6-, 12-, and 24 months of continuous culture.

### Ethics Statement

Animal approval from IACUC was not required in this study. The procedures performed on the animal were conducted as part of veterinarian standard of care procedures.

### Establishment of the Cocca-6A Cell Line

The tumor specimen was minced and treated with 0.25% trypsin under aseptic conditions to obtain a single-cell suspension, which was plated in 96-well dishes and cultured with RPMI-1640 (Hyclone, Waltham, MA) medium supplemented with 5% heat-inactivated fetal bovine serum (Hyclone, Waltham, MA), 100 lU/mL penicillin, and 1 mg/mL streptomycin (both from Hyclone, Waltham, MA). The cells were cultured and amplified in 96-, 24-, 6-well, and then in 10-cm culturing dishes. Cells were detached from the culturing dishes every three days and reseeded at a concentration of 7.5×10^5^ cells/dish. One of the clones (Cocca-6A) has been serially cultured 135 times from March 2007 to May 2011.

### Saturation Densities and Doubling Times

Logarithmically growing cell cultures were trypsinized and 5×10^4^ cells were plated in triplicate in 12-well plates (Costar, St. Louis, MO) and cultured in DMEM supplemented with 10% FBS. Cells were counted every second day by hemacytometer. The doubling time and saturation densities were calculated from a plot of cell numbers against time.

### Karyotype Analysis

Colchicine was added to logarithmically growing cell cultures, followed by an incubation for 20 min at 37°C. The cells were harvested with trypsin and incubated for 12 min at 37°C in a hypotonic solution containing 0.9% Sodium Citrate. After centrifugation, the pellet was fixed in three changes of fresh methanol-glacial acetic acid (3∶1, v/v). The fixed cells were dropped on pre-chilled (−20°C) pre-cleaned glass slides and air-dried. Chromosome spreads were “aged” at 60°C for 24 hours, subsequently stained in Giemsa for chromosome counts, and identification. A minimum of 100 spreads was evaluated for modal chromosome number.

### Cell Lines

The human embryonic kidney cell line HEK-293 was purchased from ATCC (CRL-1573) and was cultured in D-MEM supplemented with 10% FBS, L-Glutamine, Penicillin and Streptomycin all from Invitrogen Life Technologies, USA, in 95% air and 5% carbon dioxide (CO_2_) at 37.0°C. Feline Skeletal Muscle Cells (FSkMC) isolated from the limbal skeletal muscle were purchased from CellApplication (F-150-05) and were grown in Feline Skeletal Muscle Cell Growth Medium (F-151-500) (CellApplication, San Diego, CA) in 95% air and 5% carbon dioxide (CO_2_) at 37.0°C.

### Semi-quantitative Genomic PCR Analysis

Genomic DNA extraction was conducted as previously described on HEK-293, FSkMC, and Cocca-6a cells [13]. Genomic DNA was treated with RNAse-A before conducting PCR. Primers for p53 were as follows: p53-F 5′-TAC-TCC-CCT-GCC-CTC-AA-3′; p53-R 5′-GGA-GTC-TTC-CAG-TGT-GAT-GA-3′ [14]. Primers for HPRT were as follows: HPRT-F 5′-ACT-GTA-ATG-ACC-AGT-CAA-CAG-GGG-3′; HPRT-R 5′-TGT-ATC-CAA-CAC-TTC-GAG-GAG-TCC-3′. PCR reactions were conducted using the Phusion High-Fidelity DNA polymerase kit (Thermo Scientific, F-530). Annealing temperature for p53 primers was 60°C, and for HPRT primers was 65°C. The Image-J software (NIH) was used to quantify the densitometric signal acquired by a Fotodyne computerized imaging system (Fotodyne, Hartland, WI).

**Figure 2:**
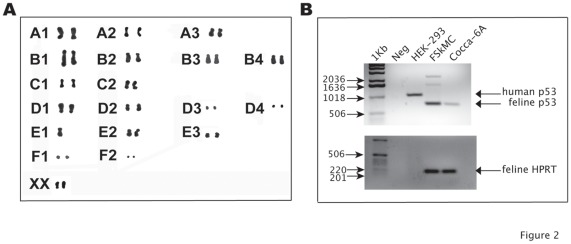
Karyogram of the feline fibrosarcoma Cocca-6A cells. (A) Oil immersion microphotograph (1000× magnification) of Giemsa block stained spotted metaphases from a representative colchicine treatment of the Cocca-6A cells. The Cocca-6A cells show the absence of one of the E1 chromosomes. (B) Semi-quantitative PCR of p53 gene in human HEK-293, feline FSkMC and Cocca 6A cells.

### Cytological Observation

The cells were examined under an inverted Olympus IX70 microscope (Olympus America, Inc. Melville, NY). Fluorescence images were captured with Sensicam QE camera (Cooke Co., Auburn Hills, MI) and managed with the SlideBook 3.0 software (Intelligent Imaging Innovations Inc., Denver, CO).

### Adenoviral Production and Purification

Ad.CMV, Ad.GFP and Ad.p53 viruses were generated using the AdEasy system (Carlsbad, CA). The Ad.RB/p105 viruses were provided by Dr. Juan Fueyo (M.D. Anderson Cancer Center, The University of Texas), the Ad.pTEN by Dr. Chris Kontos (Duke University, Durham, NC) and the Ad.p18, Ad.p19, Ad.p21^waf-1^, and Ad.p27^kip-1^ viruses by Dr. Frank L. Graham (McMaster University, Hamilton, Ontario Canada). *CTV-Mda7* (Ad.PEG-E1A-*mda-*7), CTV-IFN*-γ* (Ad.PEG-E1A-IFN*-γ*), and Ad.*mda-*7/IL-24 were provided by Dr. Paul Fisher (Virginia Commonwealth University, Richmond VA). The Ad.Ras-DN (dominant negative (116Y) v-H-RAS mutant) and Ad.p130 (RBL2/p130) viruses were purchased from Vector BioLabs (Philadelphia, PA). All the Adenoviruses were amplified, tittered and purified as previously described [15], [16]. Adenovirus transductions were performed using 50 MOI Adenoviruses (Ads), in RPMI-1640 media with 2% Fetaclone-III heat-inactivated FBS (Hyclone, Thermo Scientific, Waltham, MA). Combinations of adenoviral transductions were done using a total of 50 MOI viruses. After 16 h, the media were replaced with fresh media and cells were collected after 24- or 48-hours.

### Western Blot Analysis

Western blot analysis was conducted essentially as previously described [15], [17]. Equal amounts of protein (50 µg) were separated electrophoretically on polyacrylamide gels in the presence of SDS (SDS-PAGE) and blotted onto a nitrocellulose membrane electrophoretically. Immunodetection was performed using the enhanced chemiluminescence (ECL) system (Amersham, IL) according to the manufacturer’s instructions.

The following primary antibodies were used: mouse monoclonal antibodies against p53 (DO-1) cat#sc-126 (1∶500), p18 cat#sc-9965 (1∶100), p19 cat#sc-65594 (1∶100), RB/p105 cat#sc-102 (1∶250) (Santa Cruz Biotechnology, Santa Cruz, CA), Mda-7/IL-24 k101 (GenHunter Corporation), β-actin cat#A3853 (1∶1,500) (Sigma Aldrich); mouse polyclonal IFN-γ cat#H00003458-801 (Abnova); and rabbit polyclonal antibodies against RBL2/p130 cat#sc-317 (1∶250), p21 cat#sc-397 (1∶125), p27 cat#sc-528 (1∶500), H-Ras cat#sc-520 (1∶500) (Santa Cruz Biotechnology, Santa Cruz, CA), pTEN cat#9552 (1∶500) (Cell Signalling) and GFP cat#632377 (1∶500) (BD Bioscience).

### Flow Cytometry Assay

Media of growing cells was collected; attached cells were washed with PBS and trypsinized. Samples were prepared, run by a FACS Aria (BD Bioscience, San Jose, CA) and analyzed using the flow cytometry analysis software Flow-Jo (Flow-Jo, Ashland, OR) as previously described [17].

### Annexin-V Assay

Annexin-V was analyzed with the Annexin-V/FITC Kit (Bender MedSystems, Burlingame, CA) following manufacturer’s instructions. Cytometry was operated with a FACS Aria (BD Bioscience, San Jose, CA). Annexin-V assay experiment was repeated three times and was run as triplicate of technical repeats. Statistical analysis was performed with IBM SPSS statistic software.

### Statistical Analysis

Statistical analysis was performed using the IBM SPSS statistical software. Comparison of Sub-G1 (apoptotic fraction) populations on adenoviral transduced groups and comparison of cell death by Annexin-V on adenoviral transduced groups were conducted using an ANOVA test with post hoc test of Dunnett’s T3. *p* values of less than 0.05 were considered statistically significant.

**Figure 3:**
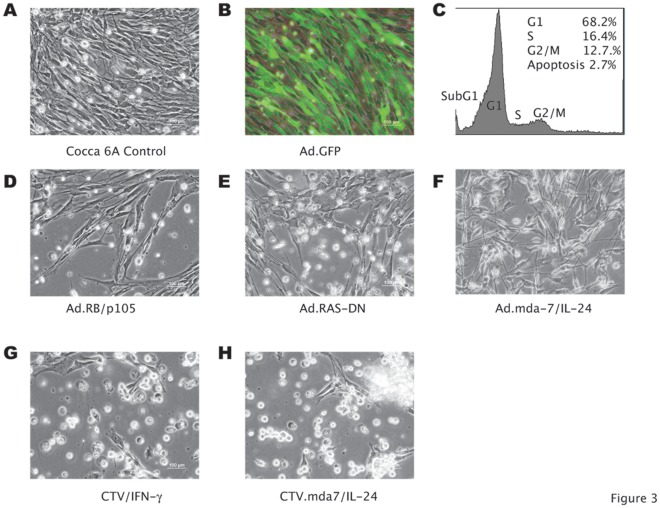
Effects on the feline fibrosarcoma Cocca-6A cells following Adenoviral gene transfer. (A) Phase contrast imaging of Ad.CMV transduced Cocca-6A control cells at 24 hours (200× magnification). (B) Fluorescence imaging (200× magnification) of the Ad.GFP-transduced Cocca-6A control cells at 24 hours. (C) Flow-cytometric diagram of Ad.GFP transduction of Cocca-6A cells (50 MOI, 24-hours after transduction). (C–H) Phase contrast imaging (200× magnification) of Ad.Rb/p105, Ad.Ras-DN, Ad.mda7/IL-24, CTV/IFNγ, and CTV-mda7/IL-24 transduced Cocca-6A cells (50 MOI, 24-hours after transduction).

**Figure 4:**
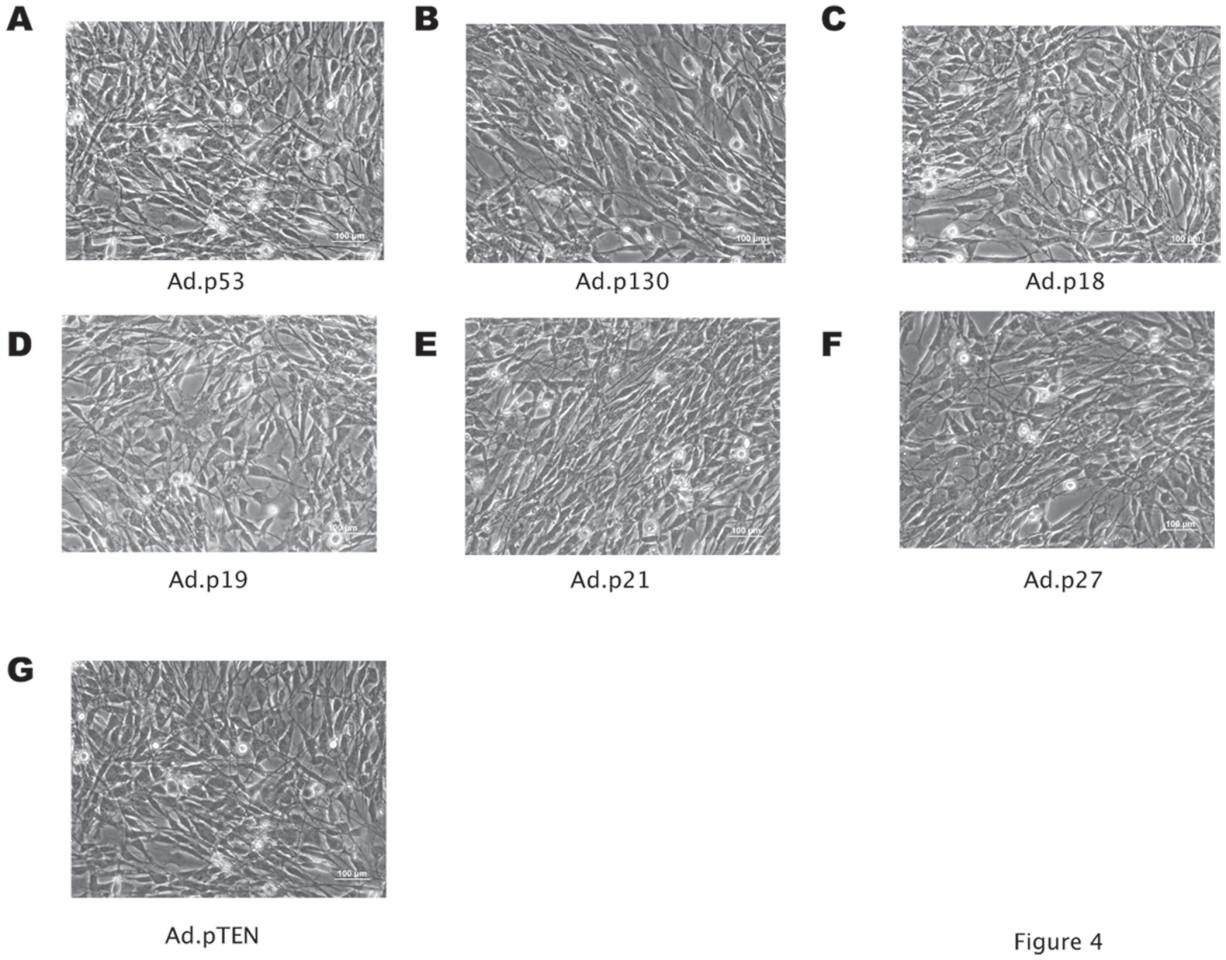
Lack of effects on the feline fibrosarcoma Cocca-6A cells following Adenoviral gene transfer. (A–G) Phase contrast imaging (200× magnification) of Ad.p53, Ad.p130, Ad.p18, Ad.p19, Ad.p21, Ad.p27, and Ad.pTEN transduced Cocca-6A cells (50 MOI, 24-hours after transduction).

**Table 1:** ANOVA (Analysis of Variance) significance table with a Post Hoc Dunnett’s T3 test for a propidium iodide flow cytometry analysis of Cocca-6A cells transduced with 50 MOIs of various single transductions and combinations of double or triple transductions using Ads carrying Ad.RB/p105, Ad.Ras-DN, Ad.mda-7/IL24, CTV.mda-7/IL24 or CTV.IFNγ.

(I) Groups	(J) Groups	MeanDifference(I–J)	Sig.
Control	CMV	.6666667	1.000
	GFP	−.8333333	1.000
	PTEN	−3.2333333	1.000
	p18	−2.6000000	1.000
	p19	.8000000	1.000
	p21	−3.6666667	.998
	p27	−4.2333333	.989
	p53	−4.5666667	.980
	RB2	−4.2666667	1.000
	RB	−25.3333333	.257
	Ras DN	−39.6000000	.135
	Ad Mda7	−18.6000000	.243
	CTV Mda7	−15.3666667	.573
	CTV IFN-γ	−18.8333333	.185
	RB + Ras DN	−21.4666667	.323
	RB + Mda7	−15.4333333	.634
	RB + CTV Mda7	−44.4666667	.345
	RB + CTV IFN-γ	−48.5666667*	.050
	Ras DN + Mda7	−14.6666667	.280
	Ras DN + CTV Mda7	−53.3666667	.057
	Ras DN + CTV IFN-γ	−52.8000000*	.011
	RB + Ras DN + Mda7	−11.1666667	.992
	RB + Ras DN + CTV Mda7	−63.9333333*	.005
	RB + Ras DN + CTV IFN-γ	−62.8666667*	.007

* The mean difference is significant at the 0.05 level.

The statistical analysis was run using IBM SPSS software.

## Results

### Establishment of a Feline Spontaneous Fibrosarcoma Cell Line

A tumor biopsy was taken from a recurring spontaneous feline fibrosarcoma, which required the amputation of the entire right limb. The specimen was sent to the pathologist for histological determination. The pathology report described the presence of an irregular dermal-subdermal mass of sheets and streams of spindle cells and small amounts of collagenous stroma, which is compatible with a diagnosis of fibrosarcoma (Fig. 1 A). Figure 1A shows at 100× enlargement the H&E staining of a histological cross-section of the feline pathologic specimen. The cells appeared to have indistinct cell borders, round to oval nuclei with finely stippled chromatin, and small single or multiple nucleoli. The cells had a mitotic index of 12/10 high power fields.

The biopsy was minced and dissociated within an hour to obtain cells for a primary culture. Cells able to attach and survive showed a typical fibroblast aspect forming various clones of the feline primary culture (Figs. 1 B–F). The clone Cocca-6A after 3 months of continuous culture started growing rapidly and the cells appeared homogeneous in size (Fig. 1 G). The morphology of the Cocca-6A cells remained unchanged after 6, 12 months (Figs. 1 H and I), still remains unchanged after 2 years and over 200 passages of continuous culture (Fig. 1 J). The Cocca-6A cells showed a serum dependent growth and did not form colonies in soft agar (data not shown). We characterized the growth of the Cocca-6A cell line and found that at serial passage 135 the cells maintained a fibroblastic appearance. Additionally, we observed that at passage one hundred, there was a population doubling time of approximately 27 hours.

### Karyotype Analysis and Semi-quantitative Genomic DNA PCR

We have analyzed the karyogram of the Cocca-6A cells at early and late passages (passages 5 and 100). Chromosome counts showed that the Cocca-6A cells were nearly diploid, with a chromosome number of 37 (normal cat; 2n = 38). Metaphase cells obtained at passage 100 had the same chromosome numbers of 37 (Fig. 2A). Chromosomes were paired following a Giemsa block staining showing the lack of one of the E1 chromosomes where, among other genes, the p53 gene product maps in feline (Fig. 2A). We have also performed a semi-quantitative genomic DNA PCR using primers against p53, which has been previously identified on chromosome E1. The p53 primers used amplified also part of the human p53 (HEK-293 cells) as shown in figure 2B. The PCR products of genomic p53 amplified from Cocca-6A cells was less abundant in comparison to the PCR product from the feline skeletal muscle cells (FSkMC). Results were normalized to the HPRT control from the X chromosomes, which amplified an equal amount of PCR product.

**Figure 5:**
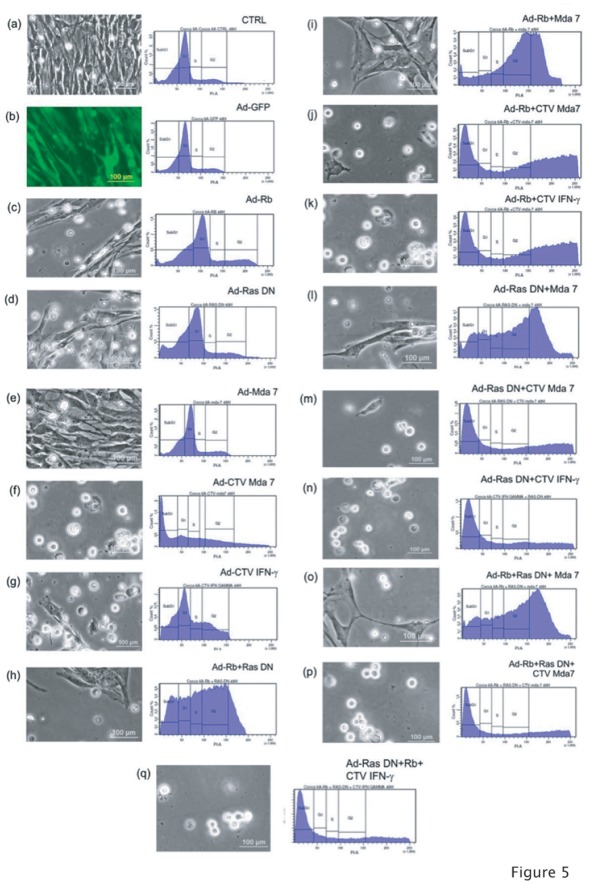
The Effects of the combination of Adenoviral gene transfer on the feline fibrosarcoma cells Cocca-6A. Every panel shows the phase contrast imaging (200× magnification, 24-hours post transduction) and the propidium iodide flow cytometry analysis of Cocca-6A cells transduced with 50 MOIs of various combinations of double or triple transductions using Ads carrying Ad.*RB/p105*, Ad.*Ras*-DN, Ad.*mda-7/IL24*, CTV.*mda-7/IL24* or CTV.*IFNγ.* (a–g) Single adenoviral transduction and controls: (a) Control untransduced Cocca-6A cells. (b) Ad.GFP, (c) Ad.Rb/p105, (d) Ad.Ras-DN, (e) Ad.Mda7/IL-24, (f) CTV.Mda7/IL-24, (g) CTV.IFNγ transduced Cocca-6A cells. (h–q) Combinations of double or triple adenoviral transductions: (h) Ad.Rb/p105+ Ad.Ras-DN, (i) Ad.Rb/p105+ Ad.Mda7/IL-24, (j) Ad.Rb/p105+ CTV.Mda7/IL-24, (k) Ad.Rb/p105+ CTV.*IFNγ,* (l) Ad.Ras-DN + Ad.Mda7/IL-24, (m) Ad.Ras-DN + CTV.Mda7/IL-24, (n) Ad.Ras-DN + CTV.*IFNγ,* (o) Ad.Rb/p105+ Ad.Ras-DN + Ad.mda7/IL-24, *(p)* Ad.Rb/p105+ Ad.Ras-DN + CTV.mda7/IL-24, (q) Ad.Rb/p105+ Ad.Ras-DN + Ad.CTV.*IFNγ* transduced Cocca-6A cells.

**Figure 6:**
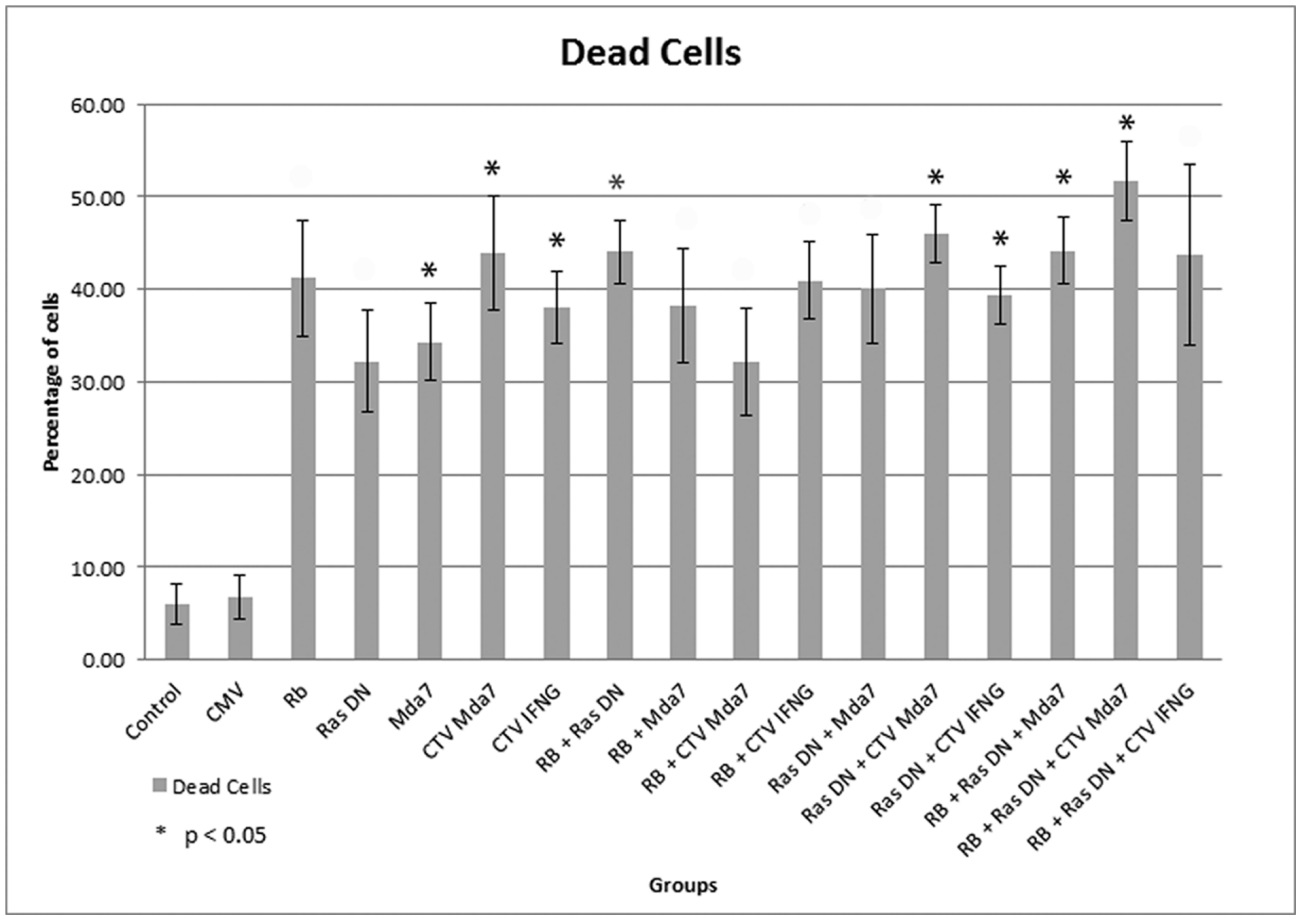
Apoptotic rate of Cocca-6A cells following Adenoviral gene transfer measured by Annexin-V assay. On the ordinate are indicated the percentages of dead cells following adenoviral transductions. On the abscissa are indicated the different adenoviruses used. Cells were stained using an Annexin-V fluorescence kit and were run on a BD scientific Facs-Aria flow cytometer.

**Table 2:** ANOVA (Analysis of Variance) significance table with a Post Hoc Dunnett’s T3 test for Annexin-V assay experiment of Cocca-6A cells following adenoviral gene transfer.

(I) Group Name	(J) Group Name	MeanDifference(I–J)	Sig.
Control	CMV	−5.46667	.585
	RB	−35.16667	.063
	Ras DN	−26.20000	.084
	Mda7	−28.30000*	.030
	CTV Mda7	−37.83333*	.050
	CTV IFN-γ	−27.33333*	.048
	RB + Ras DN	−38.00000*	.004
	RB + Mda7	−32.23333	.075
	RB + CTV Mda7	−26.10000	.099
	RB + CTV IFN-γ	−24.26667	.081
	Ras DN + Mda7	−34.00000	.055
	Ras DN + CTV Mda7	−39.96667*	.003
	Ras DN + CTV IFN-γ	−33.33333*	.005
	RB + Ras DN + Mda7	−38.10000*	.006
	RB + Ras DN + CTV Mda7	−46.93333*	.000
	RB + Ras DN + CTV IFN-γ	−37.70000	.160

* The mean difference is significant at the 0.05 level.

The statistical analysis was run using IBM SPSS software.

### Effects of Adenoviral Gene Transfer on Cat Fibrosarcoma

We have studied the effects of the transduction of various proteins involved in the cell cycle and apoptosis pathways such as Rb/p105, p130, p53, p18, p19, p21^waf-1^, p27^kip-1^, pTEN, and the dominant negative H-RAS Mutant (116Y) (RasDN), as well as of two immunostimulatory cytokines (*Mda-7/IL-24,* and *IFN-γ*) that were transferred and expressed into the feline cells using adenoviruses. Cocca-6A cells were transduced using a total of 50 MOIs of the various adenoviruses without showing a cytopathic effect. An adenovirus carrying an empty CMV promoter and Adenovirus containing the green fluorescence protein (GFP) (Fig. 3A and B) were used as controls. The cell cycle analysis of the transduced Cocca-6A cells with 50 MOIs of Ad.GFP (Fig. 3C), was comparable to the transduced Cocca-6A cells with Ad.CMV and the untransduced control (data not shown).

We observed a trend toward an increase of the sub-G1 fraction of Cocca-6A cells transduced with Ad.*RB/p105 (p = 0.257)*, Ad.*Ras*-DN (p = 0.135), Ad.Mda7/IL-24 (p = 0.243), CTV.*mda-7/IL-24 (p = 0.573)*, or CTV.*IFN*-γ (p = 0.185) (Figs. 3 D–H and Table 1), but not with Ad.*p53, Ad.p130, Ad.p18, Ad.p19, Ad.*p21^waf-1^, Ad.p27^kip-1^, or Ad.pTEN (Figs. 4 A–G and Table 1) with p>0.05, when compared to the *Ad-CMV* (Fig. 3 A and Table 1) or to Ad-*GFP* mock transduced cells (Fig. 3 B and Table 1). The transduced cells with the adenovirus carrying the green fluorescence protein (GFP) showed a normal cell cycle profile, with a small fraction of the cells in sub-G1 phase (2.7%), similar to that of Ad.CMV transduced and to untransduced cells (Fig. 3 C, Table 1, and data not shown).

Because some of the tested adenoviruses (Ads) did not elicit an effect on the feline fibrosarcoma cells (see Fig. 4) we decided to test the combinatorial effects of Ads carrying proteins that elicited a more profound response (Fig. 5 c–g and see fig. 4). A propidium iodide-flow cytometry analysis of cells transduced with 50 MOIs of various combinations of double or triple transductions using Ads carrying Ad.*RB/p105*, Ad.*Ras*-DN, Ad.*mda-7/IL24*, CTV.*mda-7/IL24* or CTV.*IFNγ* demonstrated a considerable increase of the apoptotic fraction (Sub-G1) in the various samples examined. Figure 5 depicts the contrast phase microscope images of the Cocca-6A cells and the cell cycle profiles following combined adenoviral transduction treatments, which were analyzed using an ANOVA (Analysis of Variance) with a post hoc test of Dunnett’s T3 to compare the Sub-G1 (apoptotic fraction) populations on several adenoviral transduced groups (Table 1). The following adenoviral combinations showed an increased amount of Sub-G1 population (Fig. 5h–q): Ad.Rb/p105 and Ad.Ras-DN (Fig. 5h), Ad.Rb/p105 and Ad.*mda7/IL24* (Fig. 5i), Ad.Rb/p105 and Ad.*CTV-Mda-7/IL24* (Fig. 5j), Ad.Rb/p105 and Ad-CTV IFN-γ (Fig. 5k), Ad.Ras-DN and Ad.*Mda-7/IL-24* (Fig. 5l), Ad.Ras-DN and Ad.*CTV-Mda-7/IL-24* (Fig. 5m), Ad.Ras-DN and Ad-CTV-IFN-γ (Fig. 5n), Ad.Rb/p105 and Ad.Ras-DN and Ad.*Mda-7/IL24* (Fig. 5o), Ad.Rb/p105 and Ad.Ras-DN and Ad.*CTV-Mda-7/IL24* (Fig. 5p), Ad.Rb/p105 and Ad.Ras-DN and Ad-CTV-IFN-γ (Fig. 5q).

**Figure 7:**
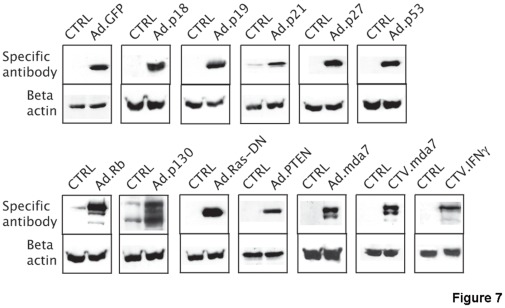
Western blot analysis of Cocca-6A cell lysates following adenoviral transduction. On the left lane are loaded control Cocca-6A cells. In the lane on right are loaded the transduced Cocca-6A cells. Anti beta-actin was used as a loading control. 50 µg of total lysates were run in SDS polyacrylamide gels. On the left side are indicated the different adenoviral transductions.

**Figure 8:**
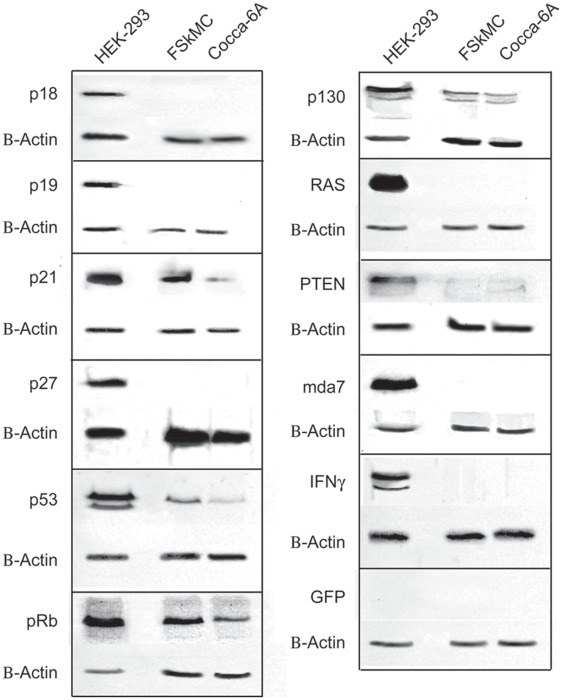
Western blot analysis of HEK-293, FSkMC and Cocca-6A cell lysates. On the left lane are loaded control human embryonic kidney HEK-293 cells and the central lane are loaded normal feline muscle skeletal cells. In the lane on right are loaded the Cocca-6A cells. Anti beta-actin was used as a loading control. 50 µg of total lysates were run in SDS polyacrylamide gels.

To quantify the apoptotic rate of the transduced cells we assayed the samples by assessing Annexin-V expression. The analyzed cells were allocated in a quadrant diagram according to their DNA content and the presence of Annexin-V on the extracellular side of the cell membrane during apoptosis. Statistical analysis was run on Annexin-V triplicates of three separate experiments using IBM SPSS statistic software for an ANOVA on dead cells with a post hoc test of Dunnett’s T3 post hoc test (Fig. 6 and Table 2). Cocca-6A cells undergoing apoptosis and necrosis were collectively called as dead cells. Ad.CMV transduced cells were compared to mock untransduced cells and no statistically difference was observed (p = 0.585, Table 2). All samples were compared to the Ad.CMV transduced negative control. Mean and standard deviation from Annexin-V experiment was calculated for each adenoviral treated groups. Groups transduced with single Ad.Mda7, Ad.CTV-Mda7 and Ad.CTV-IFNγ or double gene transfer of Ad.Rb + Ad.Ras DN, Ad.Ras DN + AdCTV.Mda7, Ad.Ras DN + Ad.CTV.IFNγ showed high statistical significant difference against the control (p<0.05, Table 2).

Cocca-6A cells treated with triple combination of Ad.RB, Ad.Ras DN and Ad.Mda7 gene transfer showed 44.13%±3.57 S.D. of dead cells with a p value of 0.006. Triple combination of Ad.RB, Ad.Ras DN and Ad.CTV-Mda7 showed the highest percentage of dead cells (51.63%±4.30S.D.) with a p value of 0.0001. Amongst the double combinational treatments, Ad.RB with Ad.Ras DN (44.03%±3.37S.D.); and Ad.Ras DN with Ad.CTV-Mda7 (46%±3.18S.D.) showed the highest percentage of dead cells. Amongst single adenoviral transductions the Ad.CTV-Mda7 (43.87%±6.17S.D.) group had the highest percentage of dead cells. Each adenoviral transduction groups showed high percentage of cell death in comparison to the control (Table 2).

Feline Skeletal Muscle Cells (FSkMC) isolated from the limbal skeletal muscle were used to investigate the effects of the various adenoviral constructs in normal cells. To quantify the apoptotic rate of the transduced normal cells we assayed the samples by assessing Annexin-V expression. Statistical analysis was run on Annexin-V triplicates of three separate experiments using IBM SPSS statistic software for an ANOVA on dead cells with a post hoc test of Dunnett’s T3 post hoc test (Fig. S1 and Table S1).

Ad.CMV transduced FSkMC cells were compared to mock untransduced FSkMC cells and no statistically difference was observed (Table S1, *p*>0.05). All samples were compared to the Ad.CMV transduced negative control. Mean and standard deviation from Annexin-V experiment was calculated for each adenoviral treated groups. All groups transduced with any of the adenoviruses carrying pro-apoptotic or cell cycle genes showed no statistical significant difference against the Ad.CMV transfected control (Table S1).

To verify the expression of the transduced adenoviral vectors, western blot analysis was performed forty-eight hours following transduction with the various adenoviral vectors. Figure 7 shows that the various adenoviruses successfully transferred and expressed the targeted transgene in the Cocca-6A cells. Antibodies against p21, pRb/p105, p130, and Beta Actin used in the western blot analysis of the transfected and untransfected Cocca-6A cells cross-reacted with feline species (control lane).

To verify the feline cross-reactivity of the antibodies, which were raised against human proteins, we performed a series of western blot analysis using human HEK-293 cells and Feline Skeletal Muscle Cells (FSkMC) isolated from the limbal skeletal muscle as controls (Figure 8). Interestingly, antibodies raised against the human p21, p53, pRb and p130 proteins cross-reacted with feline proteins from lysates of normal FSkMC cells. The p53 antibody (clone DO-1), which has been reported to cross-react with cat p53 [18], did show only a weak band in the Cocca-6A cells compared to the normal FSkMC cells (Figure 8).

## Discussion

Despite aggressive pre- or postoperative treatment, feline fibrosarcomas have high recurrence rates. Purpose of our work was to establish a cell line from a recurring spontaneous domestic cat fibrosarcoma and to test the efficiency of gene transfer for the treatment of this aggressive cancer given the lack of other therapeutic options. Spontaneous feline fibrosarcomas are more rare and have not been associated with a vaccination site [19]–[22]. A retrospective study of 195 feline sarcomas showed that 170 (87.2%) were fibrosarcomas [23]. In this work it was also noted that cats with vaccination site (VS) fibrosarcomas were younger (median  = 8 years) than cats with non-vaccination site (NVS) fibrosarcomas (median  = 11 years), but there was no such association with breed, sex, or “neuter status” [23]. The cell line we have established is derived from a spontaneous (NVS) fibrosarcoma arising on the first digit of the right front limb of a 10-year old domestic female calico cat. Although feline fibrosarcoma doesn’t appear to be an infrequent disease, no established fibrosarcoma cell lines of spontaneous origin are commercially available causing further difficulties in testing appropriate and more effective treatments for this disease.

Recently, recombinant human proteins such as Interleukin-2 (h-IL-2) has been used as a treatment option for cats with fibrosarcoma [5], [12]. It has been shown that feline fibrosarcomas treated with human IL-2 relapse less frequently and patients survive longer than control animals treated by surgery and radiotherapy alone suggesting that gene transfer approach using human gene products could be a suitable treatment option for this disease [5], [12]. In this work we have tested and compared the ability of a series of adenoviruses carrying various human proteins that are involved in regulation of the cell cycle or apoptosis such as Rb/p105, p130, p53, p18, p19, p21^waf-1^, p27^kip-1^, pTEN, and the dominant negative H-RAS Mutant (116Y) (Ras DN). We also tested the effects of two immunostimolatory human cytokines (*Mda-7/IL-24,* and *IFN-γ*) that were transferred and expressed into the feline fibrosarcoma cells using adenoviruses. Interestingly, no cell cycle modifications were observed when transducing the feline fibrosarcoma cells with single transfer of adenoviruses expressing human p53, p130, p18, p19, p21^waf-1^, p27^kip-1^, or pTEN compared to the parental control or to Ad-GFP mock transduced cells (data not shown). We have not further investigated why despite successful human protein expression in feline cells as confirmed by western blot analysis (Fig. 7), a set of cell cycle/apoptosis regulators have failed to elicit an effect on the growth and viability of the feline cells. One possible explanation is that the amino-acid sequences of the human proteins used in the adenoviral transduction assays could differ so much from the feline proteins to not be able to elicit an effect on the feline cell cycle/apoptosis machinery.

In a recent published report, the presence of numerical chromosomal and centrosomal aberrations in 5 vaccine-associated feline fibrosarcoma cell lines and in a fetal dermal fibroblast cell line as a control was determined. It was found that the number of chromosomes deviated abnormally from the normal feline chromosome number of 2n  = 38, ranging from 19 to 155 chromosomes per cell [24]. Interestingly chromosomal counts showed that the Cocca-6A cells were nearly diploid, with a chromosome number of 37 (normal cat; 2n  = 38). Metaphase cells obtained at passage 100 maintained the same chromosome numbers (Fig. 2A). Chromosomes were paired following a Giemsa block staining showing the lack of one of the E1 chromosomes (Fig. 2A). It has been previously shown that feline tumors are often associated with mutations in proteins involved in cellular proliferation control. Mutations in the p53 gene have been found associated with various tumors in people and animals including fibrosarcomas [14], [25]–[27]. The p53 gene product, that maps on feline chromosome E1 [28], is a multifunctional transcription factor that regulates induction of apoptosis in cells with irretrievably damaged DNA, thus preventing propagation of damaged DNA. More than 50% of tumors in people, including various sarcomas, have p53 gene mutations. We have shown that the fibrosarcoma Cocca-6A cells lack one of the E1 chromosomes. We performed a semi-quantitative genomic DNA PCR on the p53 gene, which is located on the E1 chromosome and have demonstrated that there is a lower amount of p53 genomic DNA in the Cocca-6A in comparison to the normal FSkMC cells.

We have tested the ability of an adenovirus carrying human p53 to modulate the cell cycle of these aggressively growing feline fibrosarcoma cells. Surprisingly, adenoviral mediated overexpression and gene replacement using human wild-type p53 did not elicit any effects in the Cocca-6A feline fibrosarcoma cell line. The lack of cell cycle effects following forced expression of human p53 in the Cocca-6A cells could be due to differences between the human and feline p53 genes, in fact in has been reported that the amino-acid sequence of the feline *p53* gene is 82.1% and 74.9% similar to the human and mouse counterparts, respectively [28].

We also tested in Cocca-6A cells the ability to induce cell cycle arrest of other cell cycle regulators, among which the Retinoblastoma protein pRb/p105, p21 (also called waf-1, CIP-1, and SDII) and p27 (also called Kip-1). The *RB/p105* gene has been found mutated in the majority of human tumors [29], [30]. Unfortunately, there is no data available regarding mutations in the *RB/p105* gene in domestic cat tumors. Interestingly, in our experiments, the expression of human RB/p105 showed relatively high levels of cell death (although not statistically significant) in the Cocca-6A feline fibrosarcoma cell line, which was instead refractory to the effects of p53.

In our quest to find the most effective gene transfer tool we also tested the effects of adenoviral transduction of a mutant form of Ras (RAS-DN) in Cocca-6A cells and found that its single expression caused high levels of cell death in the feline fibrosarcoma cell line, although not statistically significant. Ras is a protein of the GTPase family that is involved in signal transduction, cell growth and apoptosis [31]. It has been also shown that in regulating apoptosis *Ras* has many faces. It has both negative and positive effects depending on the stimulation and cell type. The responses of cells to *Ras* signaling depends on the level of *Ras* expression, the activity of various pathways, and functional cell cycle check points [32]. In fibroblasts in particular, the pathway downstream of activating Ras has been shown to involve the Raf/MAP kinase pathway, and to function in a p53 dependent manner, by increasing the levels of p16^kip-4^ and p21^waf-1^ [32]. When Cocca-6A cells were transduced with both Ad.RB and Ad.Ras DN showed higher levels of cell death in comparison to single adenoviral transduction, which were found to be statistically significant (p<0.05).

Among the other genes we transferred to the Cocca-6A cells, we tested the pro-apoptotic and tumor suppressor properties of Mda-7/IL-24. The Mda-7, Melanoma differentiation associated gene 7/IL-24, is a secreted cytokine with selective activity against cancer, with anti-apoptotic and anti-angiogenic ability. Based on sequence homology, chromosomal localization, and its functional properties, the *mda*-7 gene is now classified as a member of the IL-10 family of cytokines and named IL-24 [33]–[36]. The tumor suppressing ability of *mda*-7/IL-24 is now well established and is independent of the status of other tumor suppressor genes, such as *p53* and *Rb*, or apoptosis regulating genes, such as *bax* or *caspases*, in tumor cells [37]. Recently, a novel adenoviral vector has been created in which E1A is controlled by the PEG-3 (progression elevated-3 gene) promoter, which is a promoter that is active only in cancer cells, enabling the Adenovirus to replicate selectively in cancer cells [38]–[40]. This conditionally replicating Adenovirus is also called Cancer Terminator Virus (CTV). The CTVs have been engineered to contain sequences for anti-tumoral proteins such as Mda-7 or IFN-γ.

Transduction of the feline fibrosarcoma cells with the CTVs elicited the greatest negative effects on cellular viability (p<0.05), due to cellular combined effects of mda-7 or IFN- γ and the lytic properties of the adenoviral particles. The observed tumor cell killing effects were stronger when we transduced the feline fibrosarcoma cells with CTV-Mda7 than with IFN-γ, possibly due to the previously demonstrated Mda-7/IL-24 higher pro-apoptotic ability [37]. We speculate that for the same reason, greater cell death was observed when using a pro-apoptotic viral cocktail (Rb and Ras-DN) jointly to the immunomodulating cytokine Mda-7/IL-24.

In conclusion, we have established a spontaneous feline fibrosarcoma cell line and have identified the combinatorial use of Ad.Ras-DN, Ad.Rb and CTV-Mda7/IL-24 genes as a possible future adjuvant treatment for this case of feline fibrosarcoma. However, therapy for aggressive feline fibrosarcomas is often insufficient for complete and long lasting tumor eradication and more gene transfer studies involving various established feline fibrosarcomas should be conducted in order to understand if these viral vectors (Ras-DN, Rb, Mda7/IL24) could be applicable regardless of the origin (spontaneous vs. vaccine induced) of feline fibrosarcomas.

## Supporting Information

Figure S1 **Apoptotic rate of FSkMC cells following Adenoviral gene therapy measured by Annexin-V assay.** On the ordinate are indicated the percentages of dead cells following adenoviral transductions. On the abscissa are indicated the different adenoviruses used. Cells were stained using an Annexin-V fluorescence kit and were run on a BD scientific Facs-Aria flow cytometer.
(TIF)

Table S1 **ANOVA (Analysis of Variance) significance table with a Post Hoc Dunnett’s T3 test for Annexin-V assay experiment of FSkMC cells following adenoviral gene transfer.** The statistical analysis was run using IBM SPSS software.
(DOCX)
