# Supplementary figures and images for: Targeting a Newly Established Spontaneous Feline Fibrosarcoma Cell Line by Gene Transfer
Source: PLoS One. 2012 May 30;7(5):e37743. doi: 10.1371/journal.pone.0037743 (PMC3364269; doi:10.1371/journal.pone.0037743)

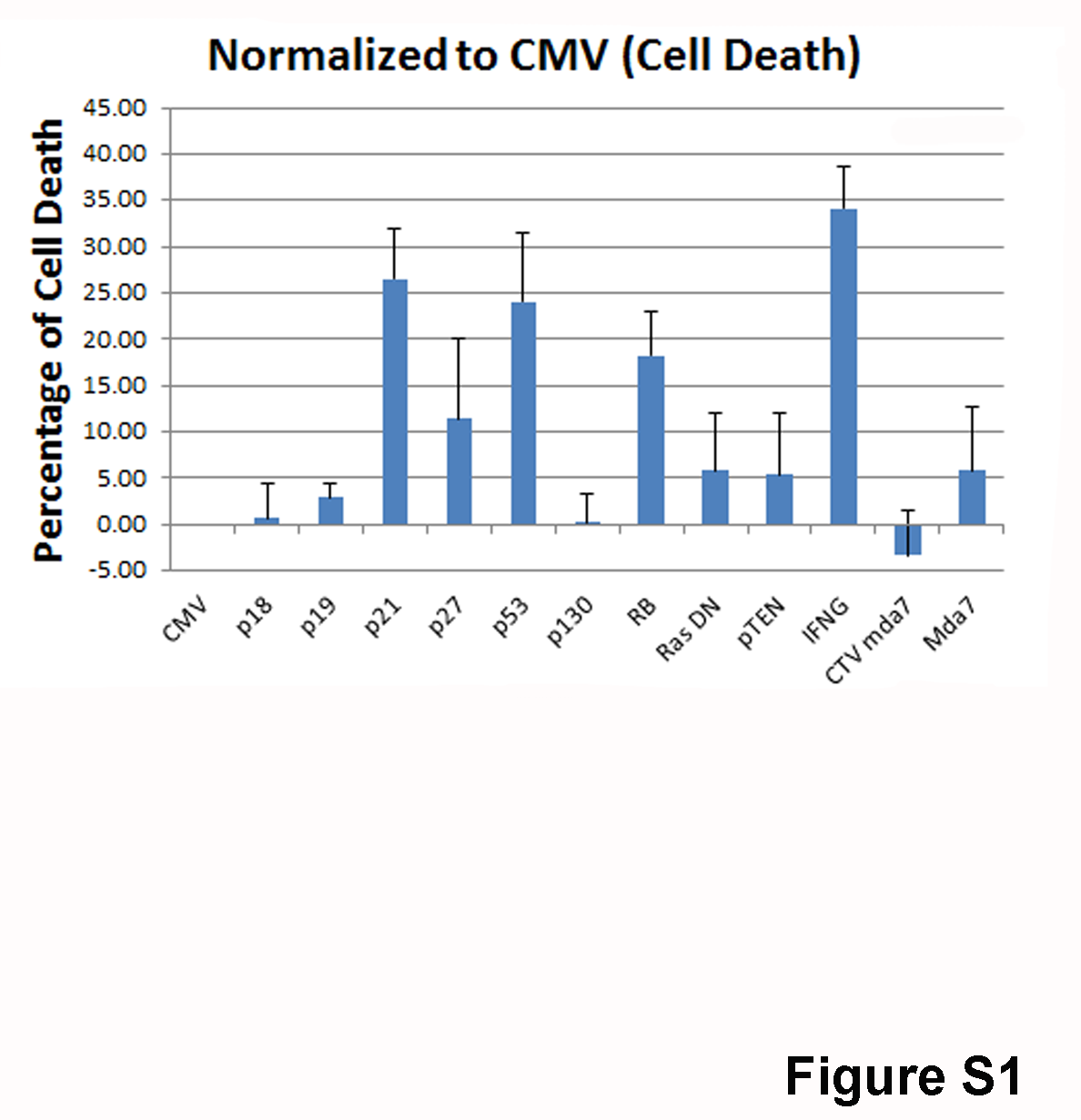

Supplement: Figure S1 — Apoptotic rate of FSkMC cells following Adenoviral gene therapy measured by Annexin-V assay. On the ordinate are indicated the percentages of dead cells following adenoviral transductions. On the abscissa are indicated the different adenoviruses used. Cells were stained using an Annexin-V fluorescence kit and were run on a BD scientific Facs-Aria flow cytometer. (TIF) [file pone.0037743.s001.tif]
