# Supplementary material for: Targeting a Newly Established Spontaneous Feline Fibrosarcoma Cell Line by Gene Transfer
Source: PLoS One. 2012 May 30;7(5):e37743. doi: 10.1371/journal.pone.0037743 (PMC3364269; doi:10.1371/journal.pone.0037743)
Supplement: Table S1 — ANOVA (Analysis of Variance) significance table with a Post Hoc Dunnett’s T3 test for Annexin-V assay experiment of FSkMC cells following adenoviral gene transfer. The statistical analysis was run using IBM SPSS software. (DOCX) [file pone.0037743.s002.docx]

**Table S1.** ANOVA (Analysis of Variance) significance table with a Post Hoc Dunnett’s T3 test for Annexin-V assay on *feline skeletal muscle FSkMC cells transduced with various adenoviruses carrying pro-apoptotic and cell cycle regulating genes.* *The statistical analysis was run using IBM SPSS software.*

| (I) Groups | (J) Groups | Mean Difference (I-J) | Sig. |
| --- | --- | --- | --- |
|  |  |  |  |
| CMV | p18 | -.70000 | 1.000 |
|  | p19 | -2.96667 | .473 |
|  | p21 | -26.50000 | .097 |
|  | p27 | -11.36667 | .733 |
|  | p53 | -24.06667 | .202 |
|  | p130 | -.20000 | 1.000 |
|  | RB | -18.23333 | .150 |
|  | Ras DN | -5.83333 | .914 |
|  | pTEN | -5.36667 | .959 |
|  | IFNG | -33.00000 | .064 |
|  | CTV Mda7 | 3.36667 | .986 |
|  | Mda7 | -5.86667 | .944 |

* The mean difference is significant at the 0.05 level.
